# Porous Al_2_O_3_-CNT Nanocomposite Membrane Produced by Spark Plasma Sintering with Tailored Microstructure and Properties for Water Treatment

**DOI:** 10.3390/nano10050845

**Published:** 2020-04-28

**Authors:** Mohamed Abdrabou Hussein, Hafiz Khurram Shahzad, Faheemuddin Patel, Muataz Ali Atieh, Nasser Al-Aqeeli, Turki Nabieh Baroud, Tahar Laoui

**Affiliations:** 1 Center of Research Excellence in Corrosion, King Fahd University of Petroleum & Minerals, Dhahran 31261, Saudi Arabia; 2 Department of Mechanical Engineering, King Fahd University of Petroleum & Minerals, Dhahran 31261, Saudi Arabia; 3 Department of Chemical Engineering, King Fahd University of Petroleum & Minerals, Dhahran 31261, Saudi Arabia

**Keywords:** membrane, carbon nanotubes, alumina, ceramic nanocomposite, spark plasma sintering, water treatment

## Abstract

Ceramic-based nanocomposite membranes are gaining great attention in various applications, such as water treatment; gas separation; oil and gas, amid their superior fouling resistance and remarkable chemical/thermal stability. Here, we report for the first time the use of spark plasma sintering (SPS) process to fabricate a porous alumina–carbon nanotubes (Al_2_O_3_–CNT) nanocomposite membrane for water treatment. The challenge is this work is to achieve a balance between the amount of porosity, desired for a high water flux, and the membrane strength level, required to resist the applied pressure during a water flow experiment. The effect of SPS process parameters (pressure, temperature, heating rate, and holding time) on the microstructure and properties of the developed membrane was investigated and correlated. A powder mixture composed of Al_2_O_3_ and 5 wt % CNT was prepared with the addition of starch as a pore former and gum Arabic and sodium dodecyl sulfate as dispersants. The powder mixture was then sintered using SPS to produce a solid but porous nanocomposite membrane. The structure and microstructure of the developed membrane were characterized using X-ray diffraction and field emission scanning electron microscopy. The performance of the membrane was assessed in terms of porosity, permeability, and mechanical properties. Moreover, the adsorption capability of the membrane was performed by evaluating its removal efficacy for cadmium (II) from water. The microstructural analysis revealed that CNT were distributed within the alumina matrix and located mainly along the grain boundaries. The permeability and strength were highly influenced by the sintering pressure and temperature, respectively. The results indicated that the membrane sintered at a pressure of 10 MPa, temperature of 1100 °C, holding time of 5 min, and heating rate of 200 °C/min exhibited the best combination of permeability and strength. This developed membrane showed a significant removal efficiency of 97% for cadmium (II) in an aqueous solution.

## 1. Introduction

Water treatment has become an essential necessity due to water shortages, and sometimes water crises, in many regions around the world. This has led to the search for and development of cost-effective technologies to address the issues related to water pollution, water reuse, and preservation of water resources [1]. Water treatment processes aim to remove undesirable constituents from water. Among the many prevailing water treatment processes, membrane filtration is considered promising and, therefore, widely accepted [2,3]. Water treatment using membrane-based separation process plays a significant role in the water treatment sector, due to its relative ease of operation, and often without chemical additives [4,5]. Ideally, membranes must have high efficiency, high stability, and low energy requirements. They should also provide a physical barrier for the “constituents of interest” based on their size. Though various categories of materials (i.e., organic and inorganic) have been utilized for membranes’ development, ceramic membranes have attracted more attention than polymeric counterparts because of their higher fouling resistance and chemical stability [5]. Although, the polymeric membrane is usually used for water treatment, due to ease of pore-forming and a relatively low cost, however, several challenges are encountered such as low resistance to fouling and low mechanical strength [6,7]. The inorganic membrane presents several advantageous properties for water treatment applications including higher resistance to chemical cleaning and wear, and better mechanical/thermal stability, thus yielding a longer service life [7].

Hydrophilic metal oxide nanoparticles, such as Al_2_O_3_ [8], SiO_2_ [9], zeolite [10], and TiO_2_ [11], are among the attractive membrane materials because of their high water permeability. Alumina has been reported to be a good absorbent [12]. In addition, incorporating nanomaterials into the membrane, improves its permeability, thermal stability, fouling resistance, and mechanical properties, as well as providing new functions like self-cleaning and contaminant degradation [2]. One example of such a nanomaterial additive is carbon nanotubes (CNT), which have been found to be attractive for heavy metal ions removal [13,14,15,16,17,18,19] and water purification applications [20,21,22,23,24,25,26,27,28,29,30,31,32,33,34] due to their unique properties, including enhanced permeability, contaminant rejection, disinfection, and antifouling behavior. Thus, the incorporation of CNT to the alumina membrane should improve its heavy metals absorption capacity. Multi-wall carbon nanotubes (MWCNT) exhibit better mechanical characteristics [35], and higher water permeability compared to Single-wall carbon nanotubes (SWCNT) [36]. Therefore, it is selected for the current study. Although the biocompatibility of CNT still remains debatable [37], it can potentially be improved with the use of CNT-based composites [38]. In composite materials’ development, the processing route is considered crucial to attaining the final material’s performance.

Various processing routes were reported for the synthesis of ceramic-based nanocomposite membranes, including conventional sintering [39,40], vibration, and pressing compaction techniques [41]. Vibration and pressing compaction methods were commonly reported for the fabrication of porous alumina and alumina–silica supports [41]. Porous alumina supports were also fabricated by conventional sintering [42]. Porous alumina that was structurally modified with CNT was synthesized by gel casting followed by high-temperature reductive sintering [43]. Porous alumina–CNT composites were also synthesized by the in-situ growth of CNTs within the porous alumina matrix via thermal pyrolysis [44].

Spark plasma sintering (SPS) is preferred over conventional sintering techniques due to its ability to apply relatively fast both pressure and temperature simultaneously which facilitate the formation of fine pores. SPS enables higher heating rate, reduced sintering time, and temperature compared to conventional sintering. Moreover, SPS yields higher strength at lower processing temperatures compared to hot pressing [45] and conventional sintering [46]. The membrane’s porosity can be manipulated by controlling the SPS temperature [47]. Therefore, the SPS technique has been reported to be promising for synthesizing porous ceramics [48]. Furthermore, improvements in the performance and reliability of such porous structures via controlling pore geometry have been reported [49,50,51,52,53]. To our knowledge, there are no reports about the synthesis of alumina–CNT nanocomposite porous membranes using SPS technique.

In the present work, we report the use of SPS to synthesize porous alumina–CNT nanocomposite membrane and the influence of SPS process parameters on the membrane’s properties, namely porosity, permeability, and mechanical strength. A nanocomposite powder consisting of alumina with 5 wt % CNT was prepared and consolidated into a porous membrane using SPS. The membrane’s properties were characterized and interrelated with the SPS parameters to obtain the best combination of membrane strength and permeability. Finally, the membrane’s potential to remove cadmium ions (Cd (II) or Cd^2+^) from water was evaluated.

## 2. Experimental Methods

### 2.1. Raw Materials and Preparation of Nanocomposite Powder

The nanocomposite powder mixture was prepared from a commercial α-alumina powder of 0.3 µm particle size (purity >95%) supplied by Buehler (Braunschweig, Germany). The purified multiwalled carbon nanotubes (MWCNT) (referred to as CNT for simplicity) with an outer diameter (OD) of 10–20 nm and a length of 10–30 µm was supplied by Times Nano (Chengdu, China). Starch (Merck, Darmstadt, Germany), was used as pore former. Polyvinyl alcohol (PVA) (Merck, Darmstadt, Germany), was used as a binder material. Gum Arabic (GA) (Scharlau, Barcelona, Spain) and sodium dodecyl sulfate (SDS) (Merck, Darmstadt, Germany), were used as dispersants.

**Figure 1:**
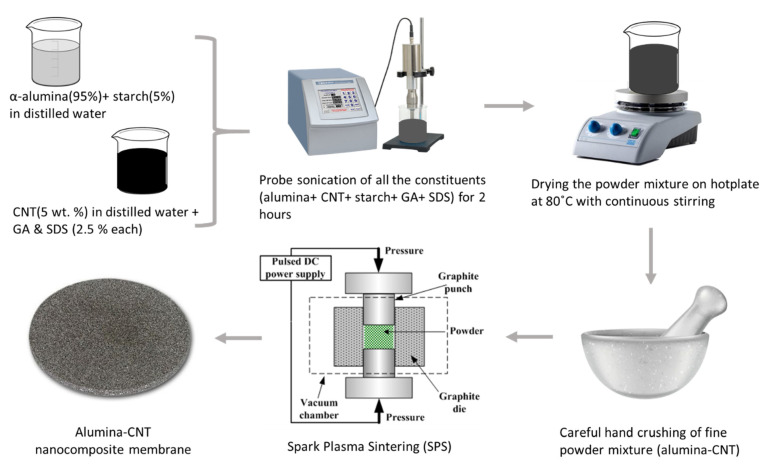
Approach and steps used for the preparation of alumina–carbon nanotubes (CNT) powder mixture and membrane.

The adopted approach to prepare alumina–CNT nanocomposite powder is illustrated in Figure 1. The first solution was prepared by adding CNT (5 wt %) to 2 liters of distilled water containing (2.5 wt %) GA and (2.5 wt %) SDS. Then, the solution was hand-mixed before being subjected to ultrasonication by probe-sonicator (Sonics, Newtown, Connecticut, USA) for 2 h. Another solution was prepared by adding a mixture of α-alumina powder (95 wt %) and (5 wt %) starch to distilled water. Both solutions were then mixed and sonicated for 2 h before being placed on a hot plate at 80 °C under continuous stirring for water evaporation. The resulting composite powder was further dried in an oven at 70 °C overnight. The dried composite powder was hand crushed to obtain a fine composite powder. To improve the compaction of the composite powder, a binder solution was prepared by dissolving 2 wt % PVA in distilled water and stirred at 80 °C until a clear solution was obtained. Finally, a 10 wt % binder solution was added to the nanocomposite powder.

### 2.2. Processing of Membrane Using Spark Plasma Sintering

Porous alumina–CNT composite membrane was fabricated using SPS machine (FCT system-model HP D5, Rauenstein, Germany) and a 30-mm graphite die. A graphite sheet was first placed inside the graphite die before adding the nanocomposite powder in order to facilitate easy removal of the sample and to reduce the friction between the die walls and the powder. The experiments were conducted in vacuum under the pressures (P) of 5, 10, or 20 MPa at the sintering temperatures (T) of 1000, 1100, or 1200 °C, heating rates (HR) of 50, 100, or 200 °C/min, and the holding times (t) of 2.5, 5, or 10 min. A parametric study was conducted to examine the influence of the SPS parameters on the membrane properties. Table 1 shows the different processing parameter sets and the assigned sample codes.

### 2.3. Characterization of Sintered Membrane

The structure and phase analysis of both as-received raw materials and SPS samples were characterized by X-ray diffraction (XRD) equipment (AXSD8, Bruker, Karlsruhe, Germany) with Cu-Kα radiation at a scanning speed of 1 degree/min. Field emission scanning electron microscopy (Lyra3, Tescan, Brno, Czech Republic), was used to study the as-received raw materials and the microstructure, particle size, and pore size of the SPS samples. The porosity of the developed membrane was measured according to ASTM C373-14a after measuring the weight of dry and wet membranes. The diametrical compression test was performed using a universal testing machine (INSTRON) to investigate the strength of the developed membrane. The diametrical strength was then calculated from Equation (1) [54,55] below:

σ = (2·*f*/π·*dt*) (1)

where *f* is the applied load, *d* is the diameter, and *t* is the thickness of the membrane.

### 2.4. Measurement of Water Flux

**Figure 2:**
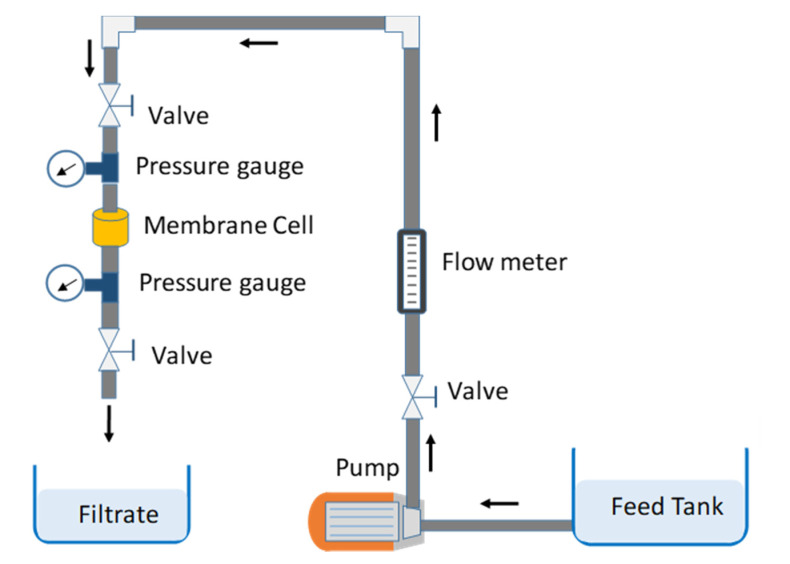
Schematic diagram of the flow loop module.

The water flux measurement was carried out using a flow loop module built in-house, as previously described (Figure 2), and the water flux J was determined using Equation (2) below:

J = *V*/*AT* (2)

where *V* is the volume of the permeate water, *A* is the effective area of the membrane, and *T* is the time required for a specific amount of water to permeate.

The water flux was measured under a transmembrane pressure of 5–40 psi. The water permeability of the membranes was calculated at transmembrane pressure P of 40 psi by dividing the water flux J with the transmembrane pressure.

### 2.5. Adsorption Capacity

The cadmium (II) (Cd (II)) adsorption capacity (R %) of the developed alumina–CNT membrane was investigated using a flow loop as depicted in Figure 2. The standard solution of cadmium (II) (1000 ppm Cd in deionized water) was prepared. The experiments were conducted in aqueous solution at room temperature and pH 6. The concentration of Cd (II) in the solution was measured by inductively coupled plasma mass spectrometry, ICP–MS (provided by Thermo Fisher Scientific, USA) before and after the experiments. Initial (*C_i_*) and final (*C_f_*) Cd ions concentrations were measured, then the adsorption capacity was calculated according to Equation (3) [56]:

*R* (%) = (*C_i_ − C_f_*)/*C_i_* (3)

## 3. Results and Discussion

### 3.1. Characterization of Raw Materials

**Figure 3:**
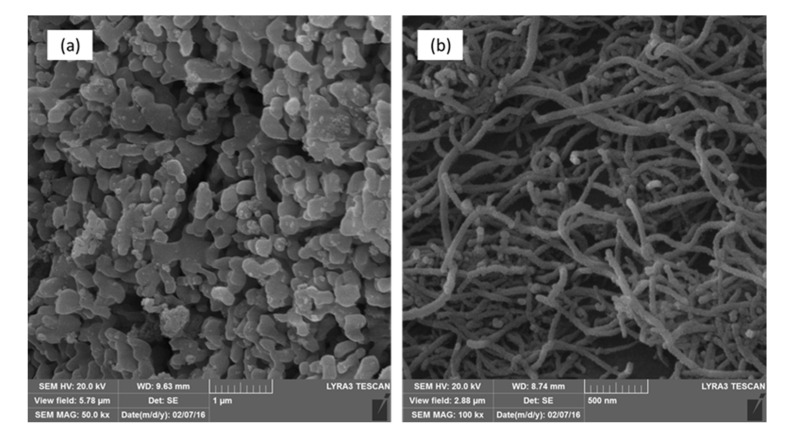
FE–SEM images of as-received (**a**) α-alumina and (**b**) CNTs.

**Figure 4:**
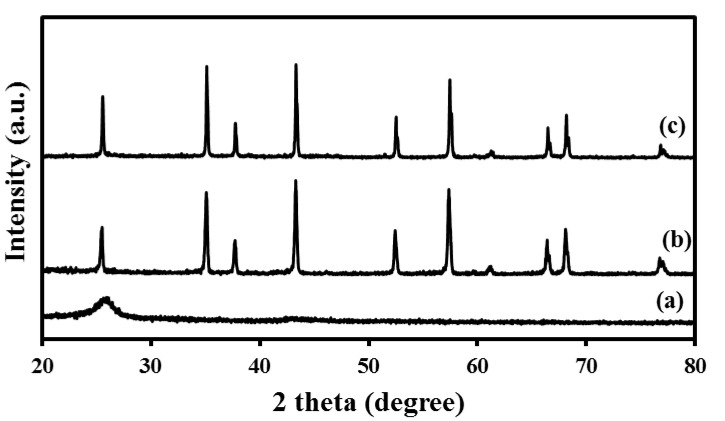
XRD patterns of (**a**) as-received CNTs, (**b**) as-received α-alumina, and (**c**) the membrane sample sintered at 10 MPa, 1100 °C, 5 min, and 200 °C/min.

The as-received materials were examined by FE–SEM and XRD to determine their structures and microstructures. FE–SEM images showed that the as-received alumina had a uniform particle size of 0.3 µm (Figure 3a), with little size variation among the particles, an important aspect for controlling the pore size. The MWCNTs had an outer diameter (OD) of 10–20 nm (Figure 3b). The XRD spectrum of the CNTs presented in Figure 4a shows two peaks corresponding to the 2-theta values of 26° and 44°, corresponding to the hexagonal graphite lattice of the MWCNTs. Figure 4b shows the XRD pattern of the as-received alumina with all the expected peaks of high-purity α-alumina.

XRD was performed, for raw as well as all the sintered membranes, to study the influence of the SPS parameters on the structure of the membranes. Figure 4c shows an XRD pattern for a selected sample (which has exhibited the best combination of strength and permeability) sintered at 10 MPa, 1100 °C, 5 min, and 200 °C/min. This spectrum contains only peaks related to crystalline α-Al_2_O_3_; the same is true for the XRD spectra of all other samples in this study (not included here). The peaks related to CNT were not even partly observed, this might be due to the small quantity of added CNT, 5 wt %, compared to the highly crystalline alumina matrix. Most importantly, no extra phases or missing peaks were noticed in any of the XRD spectra confirming that no phase changes occurred during membrane fabrication, and the final membranes consisted of the desired crystalline α-Al_2_O_3._

### 3.2. Microstructural Characterization of Spark Plasma Sintered Membranes

**Figure 5:**
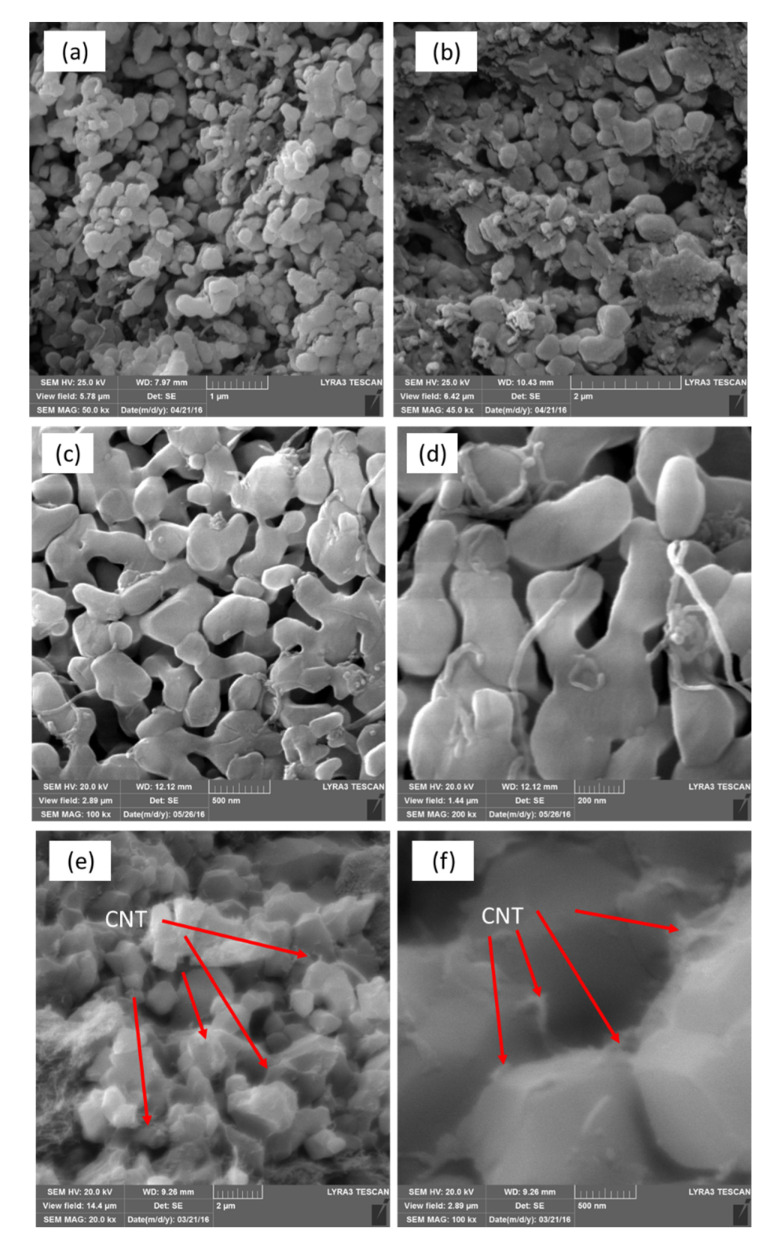
FE–SEM images of the membranes sintered at different SPS process parameters: (**a**) 5.6 MPa, 1000 °C, 10 min, 100 °C/min, and (**b**) 10 MPa, 1000 °C, 10 min, 100 °C/min; (**c**,**d**) 10 MPa, 1100 °C, 5 min, 200 °C/min; (**e**,**f**) 10 MPa, 1000 °C, 10 min, 50 °C/min.

FE–SEM was performed to investigate the microstructure and pore size of selected membrane samples. The pore size and grain growth were observed to change with increasing SPS pressure (Figure 5a,b). The membrane (Figure 5a), prepared using a sintering pressure of 5.6 MPa, showed insufficient cohesion between particles; the fine grains were very similar to those shown in the FE–SEM image of the as-received alumina (Figure 3a). Conversely, the membrane prepared using a higher pressure of 10 MPa (Figure 5b) showed relatively larger alumina particle size and a denser microstructure due to crystal growth, with alumina particles showing neck growth along adjacent particles. This growth increased with increasing sintering pressure (Figure 5a,b) and temperature (Figure 5c,d).

Figure 5e,f displays a micrograph of the fractured surface of sample SPS-4. It shows that the CNTs are well distributed within the alumina matrix and located mainly along the grain boundaries. Therefore, the nanocomposite developed in this study appears to be inter-granular [57]. Due to the simultaneous effect of pressure and temperature, SPS enables higher heating rates, shorter sintering time, and lower sintering temperature compared with conventional sintering. Hence, the utilization of SPS can potentially produce finer grains and smaller pores. Moreover, SPS could facilitate the production of higher-strength materials at lower temperatures compared with hot pressing and conventional sintering [45,46]. The effect of heating rate on the membrane porosity is shown in Figure 5e,f. At a low heating rate, the membrane demonstrates low porosity and large particles. This can be attributed to the slow sintering which in turn enables the particles to grow into larger size and subsequently decrease the amount of membrane porosity. In addition, grain shape and morphology are also affected by the low heating rate. The sintering mechanism responsible for this effect is likely to be the grain-boundary and volumetric diffusion [58,59].

### 3.3. Effect of SPS Parameters on Porosity, Water Flux, and Permeability

The permeability is the ability of the fluid to pass through the porous structure of the membrane. Both water flux and permeability depend on the porosity of the membrane. Obviously, the water flux is higher for a membrane with high porosity. The permeability depends on both water flux and transmitting pressure. The effect of SPS parameters on porosity, water flux, and permeability is presented in the following section. The porosity was measured according to the ASTM standard for ceramic materials (ASTM c373-14a) after measuring the dry and wet weights of the membrane. The water flux was measured using the flow loop as shown in Figure 2, then the permeability was calculated by dividing the water flux with the transmitting pressure.

**Figure 6:**
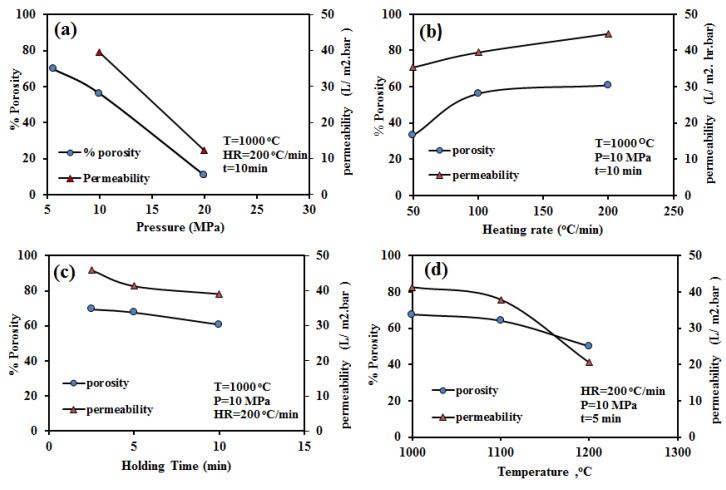
Variation in membrane porosity and permeability with SPS process parameters: (**a**) pressure, (**b**) heating rate, (**c**) holding time, and (**d**) temperature.

Figure 6 displays the change in porosity and permeability of the SPS samples with varying SPS process parameters. The porosity decreased with increasing sintering pressure (Figure 6a) and temperature (Figure 6d). However, the porosity was influenced more by the sintering pressure (decreased from 69.7% at 5.5 MPa to 10.77% at 20 MPa) than the sintering temperature (67% at 1000 °C and 50% at 1200 °C). The elevated values of temperature and pressure contributed to the neck growth of the alumina particles and thus reduced the porosity. The observed decrease in porosity with increasing sintering pressure and temperature might be due to the sintering of adjacent particles. However, the porosity increased with the increase in heating rate (Figure 6b) (33% at 50 °C/min to 69% at 200 °C/min). The holding time demonstrated a similar correlation with membrane porosity. Notably, with the increase in the holding time from 2.5 min to 10 min, the porosity decreased from 69.3% to 60.7% (Figure 6c).

**Figure 7:**
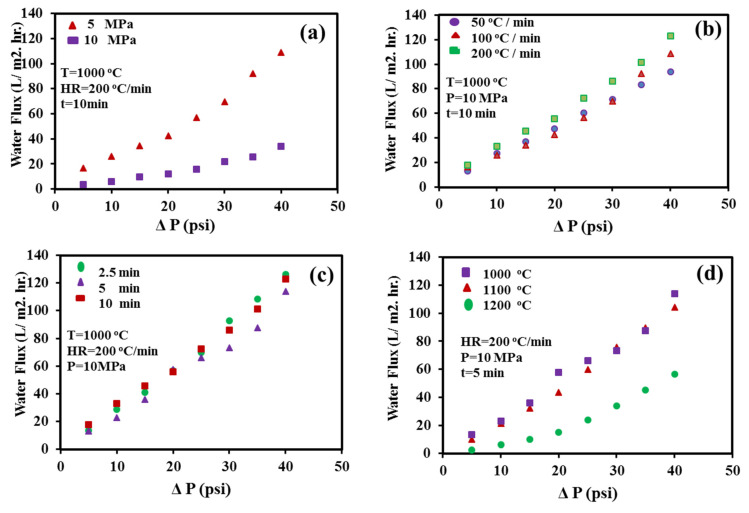
Variation in pure water flux under different transmittance pressures for membranes prepared with varying SPS process parameters: (**a**) pressure, (**b**) heating rate, (**c**) holding time, and (**d**) temperature.

The water flux through the sintered samples was measured at a transmembrane pressure of 5–40 psi using the setup shown in Figure 2. Water flux was observed to increase with increasing transmittance pressure for all conditions (Figure 7). The water flux was found to be more influenced by the SPS pressure and temperature than by heating rate or holding time, which could be attributed to the porosity. Although the sample that was compacted at 5.6 MPa possessed the highest porosity compared to other samples, the sample broke during the water flux test. Thus, this compaction pressure yielded a membrane with low strength and consequently did not produce a sufficiently robust membrane for practical application. The membrane permeability for different SPS processing conditions, representing the membrane’s productivity, was calculated from the water flux measurement. 

**Figure 8:**
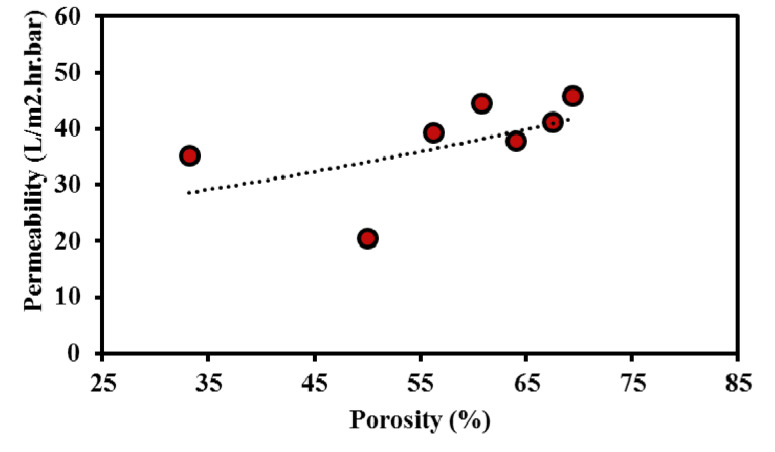
Variation of permeability with porosity level for different porous membranes.

As shown in Figure 6, the membrane permeability follows a similar trend to that of the porosity for the different processing parameters: it decreases with increasing sintering temperature (decreased from 41.58 L/m^2^·hr·bar at 1000 °C to 20.53 L/m^2^·hr·bar at 1200 °C). However, it increases with increasing heating rate (increased from 35.3 L/m^2^·hr·bar at 50 °C/min to 44.5 L/m^2^·hr·bar at 200 °C/min). The highest permeability values of 45.86 and 44.5 L/m^2^·hr·bar is obtained for the samples sintered at (10 MPa, 1000 °C, 2.5 min, and 200 °C/min; SPS-7), and (10 MPa, 1000 °C, 10 min, and 200 °C/min; SPS-5), respectively. Figure 8 depicts the permeability as a function of porosity, obtained from porous membranes processed under different process conditions. As expected, the permeability increases by increasing the percentage of porosity, in agreement with data reported in the literature [60].

### 3.4. Mechanical Properties of Al_2_O_3_–CNT Nanocomposite Membrane

**Figure 9:**
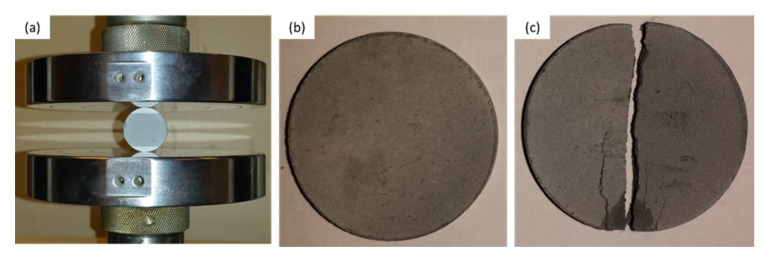
Diametrical compression testing: (**a**) configuration, (**b**) membrane sample before the test, and (**c**) fractured membrane after the test.

Diametrical compression tests were carried out to evaluate the strength of each membrane (Figure 9). In each case, the test was completed when the sample fractured into two halves (Figure 9c) because of the tensile failure [40]. Increasing the sintering temperature from 1000 °C to 1200 °C resulted in an increase in the diametrical strength of the SPS membrane from 1.9 MPa to 12.3 MPa (Table 1), a change that could be attributed to grain growth and pore shrinkage. Similar results were obtained with increasing pressure SPS-1 sintered at a pressure of 20 MPa exhibited a strength of 12.3 MPa, whereas SPS-3 sintered at a pressure of 5.6 MPa showed a strength of 1.92 MPa (Table 1). These findings reveal that SPS both temperature and pressure have a significant effect on the strength of the membrane. High pressure brings particles closer together during compaction, and high temperature causes them to diffuse with one another easily; this result is consistent with previous research [39]. Keeping the other parameters constant, the strength increases with increasing sintering pressure, which can be attributed to an increase in the densification of the membrane. This increase in densification reflects an increase in interface formation, which ensures effective load sharing between the matrix (Al_2_O_3_) and filler (CNT), as confirmed by the FE–SEM micrograph shown in Figure 5; these results are also consistent with previous studies [39,61]. In contrast, the heating rate displays an antagonistic effect on strength: an increase in the heating rate from 50 °C/min to 200 °C/min causes the membrane’s strength to decrease from 8 MPa to 4.9 MPa. This reduction in strength can be attributed to a higher amount of porosity, accompanied by a poor load sharing ability. 

### 3.5. Correlation between Permeability and Strength of Al_2_O_3_-CNT Membrane

**Figure 10:**
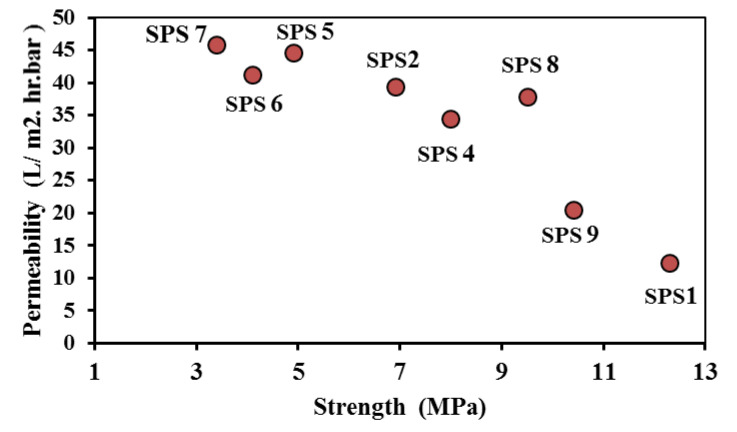
Permeability and strength of alumina–CNT membranes produced under different SPS process conditions.

It is important to analyze and correlate the properties of the developed Al_2_O_3_-CNT nanocomposite membrane with the processing parameters to obtain the best combination in terms of permeability and strength. The permeability is related to the functionality of the membrane, while its strength plays a significant role in its reliability and service life [62]. The permeability and strength of the membranes produced under different SPS conditions (Figure 10) were analyzed to identify the best combination. Membrane SPS-8 showed the optimal combination, followed by membranes SPS-4 and SPS-2. The average pore size was calculated using the FE–SEM micrographs. For SPS-8, the average value of pore size was 0.14 ± 0.04 µm and ranged from 0.08 µm to 0.24 µm. The formation of the pores is attributed to both the burning off the pore former and partial sintering [59,63].

### 3.6. Adsorption Capacity of Cadmium (II) on Al_2_O_3_–CNT Nanocomposite Membrane

To test the capability of the developed membrane for heavy metals removal, the adsorption capacity of cadmium (II) (or Cd (II)) from water was performed on the selected membrane (SPS-8). Adsorption capacity of Cd (II) from water was carried out using the flow loop shown in Figure 2 at pH = 6 to avoid precipitation of Cd (II) as reported in our previous work [55]. The developed membrane showed a removal efficiency of 97% for Cd (II), close to the values reported in the literature for other membranes [13,64,65] as presented in Table 2. Moreover, it showed a higher removal efficiency compared to either α-alumina or alumina–CNT powder mixture tested in similar conditions [55]. The mechanism of metal ions attachment on CNT is sorption and electrostatic attraction [66], while for alumina, it is simply physical adsorption [67]. Therefore, the addition of CNT, as a filler, in the alumina matrix has improved the adsorption/removal efficiency. CNT are reported to possess a great potential for the removal of various heavy metals ions from wastewater such as Cd [68,69], Cr, Cu, and Ni [69].

## 4. Conclusions

A porous alumina–carbon nanotubes (Al_2_O_3_–CNT) nanocomposite membrane was fabricated by spark plasma sintering (SPS). The microstructural analysis revealed that CNT were distributed within the alumina matrix and located mainly along the grain boundaries. By varying the SPS process parameters (pressure, temperature, heating rate, and holding time), the best combination of strength and membrane permeability was achieved. Overall, the SPS sintering pressure and temperature were found to be more influential factors in controlling the properties of the membrane compared to other factors such as heating rate and holding time. The porosity was influenced more by the applied pressure, followed by the sintering temperature. The strength, water flux, and permeability of the membrane were more influenced by the sintering temperature, followed by the applied pressure. The membrane sintered at 10 MPa pressure, 1100 °C temperature, 5 min holding time, and 200 °C/min heating rate revealed the best combination of permeability (37.8 L/m^2^·hr·bar), and strength (9.5 MP), along with an average pore size of 0.14 µm. Furthermore, this developed membrane was able to remove 97% for cadmium (II) in an aqueous solution.

The present study has allowed to delineate the impact of SPS parameters on the porous Al_2_O_3_–CNT membrane’s properties and performance, applied here in water treatment, but could ultimately pave the way for more practical applications of ceramic membranes.

## Figures and Tables

**Table 1:** Spark plasma sintering (SPS) processing parameters, porosity, diametrical strength, and permeability of SPS samples.

Processing Parameters					Experimental Results		
Sample Code	Temperature (°C)	Time (min)	Heating Rate (°C/min)	Pressure (MPa)	Porosity (%)	Diametrical Strength (MPa)	Permeability (L/m^2^·hr·bar)
SPS-1	1000	10	100	20	10.77	12.3	12.31
SPS-2	1000	10	100	10	56.2	6.9	39.45
SPS-3	1000	10	100	5.6	69.7	1.9	NA
SPS-4	1000	10	50	10	33.2	8	35.34
SPS-5	1000	10	200	10	60.7	4.9	44.58
SPS-6	1000	5	200	10	67.5	4.1	41.32
SPS-7	1000	2.5	200	10	69.3	3.4	45.86
SPS-8	1100	5	200	10	64	9.5	37.89
SPS-9	1200	5	200	10	50	10.4	20.53

**Table 2:** Removal efficiency of Cd (II) by different membranes as reported in the literature.

Membrane	Removal Efficiency (%)	Reference
Alumina–CNT membrane	97	This work
Modified CNT	93	[13]
α-alumina	28	[55]
Alumina–CNT powder mixture	79	[55]
Polysulfone	92–98	[64]
Amicon regenerated cellulose	99	[65]
